# Antimicrobial stewardship of Chinese ministry of health reduces multidrug-resistant organism isolates in critically ill patients: a pre-post study from a single center

**DOI:** 10.1186/s12879-016-2051-8

**Published:** 2016-11-25

**Authors:** Xudong Ma, Jianfeng Xie, Yi Yang, Fengmei Guo, Zhiwei Gao, Hua Shao, Yingzi Huang, Congshan Yang, Haibo Qiu

**Affiliations:** 1 School of Medicine and Health Management, Tongji Medical College of Huazhong University of Science and Technology, Wuhan, 430074 China; 2 Department of Critical Care Medicine, Nanjing Zhongda Hospital, School of Medicine, Southeast University, Nanjing, 210009 China; 3 Department of Pharmacy, Nanjing Zhongda Hospital, School of Medicine, Southeast University, Nanjing, 210009 China

**Keywords:** Antimicrobial stewardship, Antimicrobial consumption, Defined daily dose, Multidrug-resistant organism

## Abstract

**Background:**

China’s Ministry of Health (MOH) has established a policy about the antimicrobial stewardship. To date, the effects of this policy on multidrug-resistant organism (MDRO) in critically ill patients are unknown.

**Methods:**

A pre-post study was conducted on intensive care unit (ICU) patients from June 2010 to May 2011 and from June 2012 to May 2013. Bacterial cultures were conducted at ICU admission and discharge. In June 2011, our hospital started to administer the antimicrobial stewardship program of Chinese MOH. We collected the data on antimicrobial consumption during the 3-year period in all hospital and individual department every month, and analyzed the correlation between the proportion of critically patients colonized or infected with MDRO and antimicrobial consumption.

**Results:**

A total of 978 patients were involved in the present study. With the intervention, the monthly mean Defined Daily Dose (DDD) per 100 occupied bed-days throughout the hospital decreased from 96 ± 7 to 65 ± 6 (*p* < 0.001), and the proportion of patients colonized or infected with MDRO decreased from 36 to 13% at the time of ICU admission and declined from 48 to 29% at the time of ICU discharge (both *p* < 0.001). There was a significant positive relationship between the proportion of all critically ill patients colonized or infected with MDRO at ICU admission and the DDD of the entire hospital (R^2^ = 0.7858, *p* < 0.001).

**Conclusion:**

The antimicrobial stewardship program of Chinese MOH reduced the consumption of antibiotics. Moreover, the proportion of patients colonized or infected with MDRO decreased along with reduced consumption of antibiotics.

**Trial registration:**

Retrospectively registered: NCT02128399; Date of registration: 22 APR 2014; Detail information web link: https://clinicaltrials.gov/ct2/show/NCT02128399?term=NCT02128399&rank=1

**Electronic supplementary material:**

The online version of this article (doi:10.1186/s12879-016-2051-8) contains supplementary material, which is available to authorized users.

## Background

Antimicrobial exposure increases the selection for various drug-resistant organisms [1]. Numerous studies have demonstrated the association between antimicrobial use and MDRO detection [2–4]. However, to our knowledge, there are few data to support the concept that reducing antibiotic use actually leads to improvements in antibiotic susceptibilities [1]. Moreover, antimicrobial stewardship was demonstrated to reduce MDRO and was strongly recommended in clinics [5]. The World Health Organization (WHO) strongly recommends that governments implement antimicrobial stewardship programs directed at containing antimicrobial resistance [6]. However, a considerable gap exists between theory and clinical practice [7]. Antibiotic use is increasing even in some countries such as Norwegian with a low antibiotic resistance rate [8, 9].

In China, the overuse of antibiotics is common [10], and the prevalence of antimicrobial resistance isolates is higher than that in North America and Europe [11]. Data reported by the Chinese Ministry of Health (MOH) National Antimicrobial Resistance Investigation Net (Mohnarin) indicates that antimicrobial resistance is rising steadily [12]. In 2011, Chinese MOH established policies concerning the antimicrobial stewardship to improve the intelligent use of antibiotics. Most hospitals in China gradually implemented the policies. These policies included restricting the kinds of antibiotics, setting the targets for antibiotic prescription in hospitalized patient and prophylactic use of antibiotics in clean operations (Please see the detail of polices in methods of *Antimicrobial stewardship*). However, to the best of our knowledge, no studies have shown whether the antimicrobial stewardship can reduce infection or colonization with MDRO in critically ill patients. Therefore, in the present study, we aimed to elucidate the role of the antimicrobial stewardship on the antimicrobial consumption and infection or colonization with MDRO in critically ill patients.

## Methods

### Study design

The pre-post study was performed in the ICU of a university-affiliated hospital. The protocol was approved by Nanjing Zhongda Hospital Institutional Ethics Committee (Approval Number: 2011ZDLL012.0). Written informed consent was obtained from the patients or next of kin. The trial was registered at clinicaltrials.gov (NCT02128399).

The study was divided into two periods according to the intervention of antibiotics management policy, which started in June 2011. Phase 1 was defined as one year before the intervention (from June 2010 to May 2011). Phase 2 was defined as one year after the intervention (from June 2012 to May 2013). During the two phases of the study, all patients hospitalized in the ICU and patients discharged from ICU before the end of the two studied phases were eligible to participate in the study. We only selected the data of patients for their first admission to ICU. Screening for MDRO was performed in our ICU as part of an infection control policy, and microbiological cultures of different specimens were performed according to clinical status. Bacterial cultures from na**s**al swabs, lower respiratory tract secretions and the infected area were performed at the time of ICU admission and discharge.

We performed the same policy of screen during the two study periods. We also had taken executive infection control measures during this pre-post study period in our hospital. All of the patients were treated by the attending physician.

### Antimicrobial stewardship

According to the protocol of antibiotic administrative group, our hospital was directed to implement the protocol in June 2011. The policy included antibiotic procurement was restricted to 50 agents in our hospital from June 2011. Meanwhile, targets for antibiotic prescription are set at less than 60 and 20% of all prescriptions for hospitalized patients and outpatients, respectively. Prophylactic use of antibiotics in clean operations should be lowered to 30% of patients and reduced to less than 24 h’ duration; and antibiotic utilization in hospitalized patients should be less than 40 Defined Daily Dose (DDD) per 100 patient; rationality of antibiotic use, which includes timing, duration and appropriate medications should be more than 90%. In our hospital, special antimicrobial agents, such as carbapenem, glycopeptide, linezolid, daptomycin, and tigecycline, must be approved by a designated doctor of pharmacy; in particular case, such as during salvage, doctors are permitted to urgently use these special antimicrobial no more than one day. The trained pharmacists and infection preventionists were all part of the antimicrobial stewardship in our hospital. They were responsible for monitoring these medications, and every month, they announced the antimicrobial agents in every clinic and department. If the individual department did not reach the targets which mentioned, the director of the department would provide the reason and be warned by the hospital. If there was no desirable reason why they could not achieve the targets, the salary of all the staff of the department would be deducted. During the study period, we practiced infection control measures according to the recommendations of the Centers for Disease Control (CDC) of the USA [13].

### Data collection and definitions

Beginning in June 2011, the management of antibacterial drugs in clinical applications was implemented in every department in our hospital. For each month, we recorded the aggregate data on active surveillance testing and antibiotic consumption. We prospectively collected all data concerning patient characteristics at the time of ICU admission and during the patients’ ICU stay. We collected information about ICU patients’ microbiological culture results and antibiotic consumption between June 2010 and the end of May 2011. The antibiotic consumption data were obtained from the hospital computer center database.

We used the Defined Daily Dose DDD per 100 occupied bed-days to indicate antibiotic consumption in our hospital and every department. The DDD for adults was obtained from the anatomical therapeutic chemical (ATC) classification index from the WHO, with the DDD unit expressed in grams.

MDRO were defined as bacteria were resistant to at least three antimicrobial classes which included methicillin-resistant Staphylococcus aureus (MRSA), vancomycin-resistant enterococci (VRE), Pseudomonas aeruginosa, Acinetobacter baumannii, and extended-spectrum β-lactamase (ESBL)-producing or carbapenemase-producing gram-negative bacilli [14]. We use laboratory tests to determine these MDRO. Minimal Inhibitory Concentration (MIC) method was used to determine the bacterial drug resistance. MRSA was defined as Staphylococcus aureus resistant to oxacillin and VRE was defined as enterococci resistant to vancomycin. ESBL-producing organisms were defined as Gram-negative bacilli resistant to ceftazidime but sensitive to enzyme inhibitor such as piperacillin–tazobactam.

#### Statistical analysis

All of the statistical analyses were conducted using SPSS 13.0 software (release 13.0, SPSS, Chicago, IL, USA). The distribution of quantitative variables was tested. Normally and abnormally distributed quantitative variables are presented as the mean ± standard deviation and the median (25th–75th interquartile range), respectively. All *p* values were two-tailed. A *p* < 0.05 was considered to be statistically significant.

We assessed the differences in categorical variables using the *χ* ^2^ test or Fisher’s exact test. We analyzed the differences in length of ICU stay and stay until hospital discharge or death using the Mann–Whitney *U* test.

Antibiotic consumption and infection or colonization with MDRO were assumed to be independent variables; the association between the 2 variables was evaluated using the parametric Pearson’s or non-parametric Spearman’s correlation coefficient.

## Results

### General condition

A total of 978 patients were involved in our study, among whom 433 patients were involved before the intervention and 545 patients were involved after the antibacterial drug clinical application management. The patient characteristics are presented in Table 1. Of the 978 patients, the median length of the ICU stay was 5 (2–10) days, and the median length of the hospital stay was 18 (8–30) days. One hundred fifty-six (24%) patients died in the ICU, and the hospital mortality rate was 26% (Table 1). There were no significant differences in ICU mortality, hospital mortality and length of ICU stay between both phases; however, the length of the hospital stay was reduced after the intervention (17 days vs. 18 days, *p* = 0.009).

**Table 1:** Summary of patient characteristics

	All patients (978)	Before management (433)	After Management (545)	*p* value
Age, years	64 ± 19	64 ± 19	65 ± 18	0.319
Male (%)	630 (64)	267 (62)	363 (67)	0.109
APACHEII score	16 ± 9	16 ± 9	16 ± 9	0.578
Admission source				
Emergency	313 (32)	132 (30)	181 (33)	0.268
Surgery	473 (48)	205 (47)	268 (49)	0.569
Medicine	192 (20)	96 (22)	96 (18)	0.075
ICU mortality (%)	244 (25)	112 (26)	132 (24)	0.55
Hospital mortality (%)	281 (29)	133 (31)	148 (27)	0.22
Duration of ICU stay	5 (2–10)	5 (2–10)	5 (2–10)	0.954
Duration of hospitalization	17 (8–30)	18 (8–33)	17 (9–30)	0.009

*APACHEII* Acute Physiology and Chronic Health Evaluation II

### Antimicrobial consumption

**Fig. 1:**
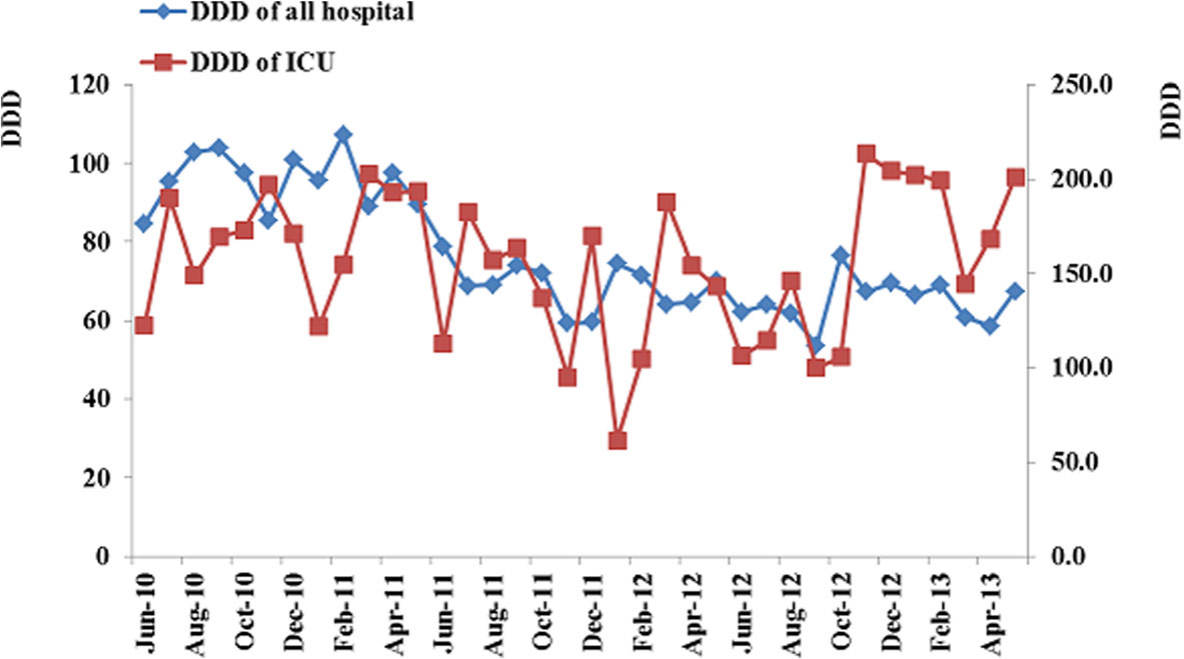
Antibiotic consumption of our hospital and ICU decreased after the management of antibacterial drugs in clinical applications

The monthly antimicrobial consumption throughout the hospital from June 2010 to May 2013 is shown in Fig. 1. With the antimicrobial stewardship program in clinical applications, we found that the rational use of antibiotics had improved. In the preventative use of antibiotics in clean operation, for example, the percentage decreased from nearly 100 to 35%. The DDD per 100 occupied bed-days in our hospital decreased from 96 before intervention to 65 after intervention (Table 2). In most departments, the DDD per 100 occupied bed-days decreased similarly, and only in the thoracic surgery, neurology and urology departments, the DDD per 100 occupied bed-days did not decrease from 2010 to 2013 (Table 2).

**Table 2:** Mean monthly DDD per 100 occupied bed-days before and after the antimicrobial stewardship

Department	Before management	After management	*p* value
All	96 ± 7	65 ± 6	<0.001
Neurosurgery	78 ± 26	45 ± 17	0.002
Thoracic surgery	96 ± 20	106 ± 36	0.38
General surgery	115 ± 16	93 + 20	0.003
Urology surgery	117 ± 15	129 ± 10	0.02
Orthopedics	82 ± 9	40 + 7	<0.001
Respiratory medicine	178 ± 18	128 + 15	<0.001
Neurology	36 ± 9	35 ± 12	0.963
Cardiology	74 ± 12	35 ± 6	<0.001
ICU	170 ± 28	159 ± 44	0.018

The monthly antimicrobial consumption in the ICU is shown in Fig. 1. The DDD per 100 occupied bed-days in the ICU during the study period was higher than that observed in other departments. With the antimicrobial stewardship, the DDD per 100 occupied bed-days also decreased from 170 ± 28 to 159 ± 44 in ICU (*p* = 0.018).

During the antimicrobial stewardship, the category of antibiotics did not change (Additional file 1: Table S1). The median duration of antibiotics were 3.64 (1.96, 6.18) and 3.42 (1.65, 6.19) before and after antimicrobial stewardship, respectively. In addition, the dose of antibiotics also did not change during the intervention.

### Relevance ratio of multidrug-resistant organisms

**Fig. 2:**
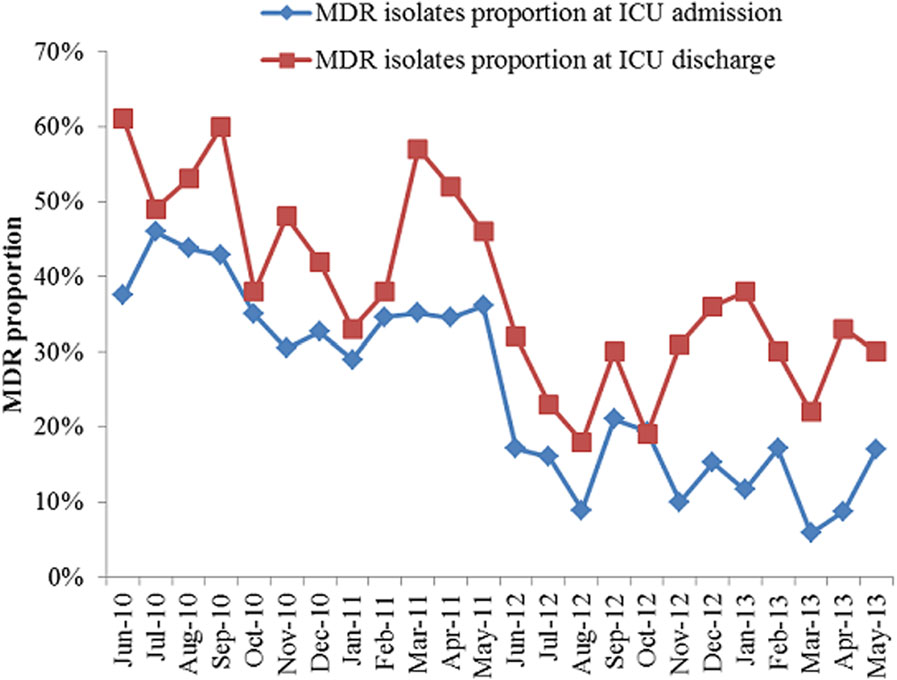
Proportion of patient colonization of infection with MDRO at the time of ICU admission and discharge decreased after antimicrobial clinical application management

The proportion of colonization and infection with MDRO in critically ill patients at the time of ICU admission and ICU discharge every month is shown in Fig. 2. Among the total patients included in the study, the proportion of MDRO isolates decreased from 36 to 13% at the time of ICU admission and declined from 48 to 29% at the time of ICU discharge (both *p* < 0.001). Proportion of MDRO isolates of patients who admitted into ICU directly from emergency department, which indicate that patients directly colonized from the community, decreased from 40 to 18% (*p* < 0.001). Proportion of MDRO isolates of patients who transferred into ICU form other wards decrease from 34 to 11% (*p* < 0.001). However, the proportion of MDRO isolates in patients from different departments decreased in varying degrees, even in some departments in which DDD was not decreased, such as in the thoracic surgery department (Table 3).

**Table 3:** Proportion of patients colonized or infected with MDRO before and after antimicrobial stewardship

Department	All patients (978)	Before management (433)	After management (545)	*p* value
All, n (%)	225 (23.01)	154 (35.57)	71 (13.03)	<0.001
Emergency, n (%)	86 (23.76)	54 (40.91)	32 (17.68)	<0.001
Surgery, n (%)	91 (19.24)	68 (33.17)	23 (8.58)	<0.001
Neurosurgery, n (%)	11 (13.75)	5 (29.41)	6 (9.52)	0.035
Thoracic surgery, n (%)	12 (10.52)	11 (20.37)	1 (1.67)	0.001
General surgery, n (%)	35 (26.92)	28 (40.00)	7 (11.67)	<0.001
Urology surgery, n (%)	10 (28.57)	8 (38.10)	2 (14.29)	0.252
Orthopedics, n (%)	8 (18.18)	8 (40.00)	0 (0)	0.001
Other, n (%)	13 (18.57)	6 (26.09)	7 (14.89)	0.258
Medicine, n (%)	50 (27.47)	34 (30.52)	16 (16.84)	0.033
Respiratory medicine, n (%)	10 (32.26)	8 (50.00)	2 (13.33)	0.054
Neurology, n (%)	13 (38.23)	8 (42.11)	5 (33.33)	0.728
Cardiology, n (%)	7 (15.22)	5 (21.73)	2 (8.70)	0.218
Other, n (%)	20 (25.00)	13 (35.14)	7 (16.28)	0.062

There were 802 and 992 samples for culture in phase 1 and phase 2 at ICU admission, respectively. Table 4 displays the microorganisms associated with colonization or infection at the time of ICU admission and ICU discharge. Among the 273 MDRO present at the time of ICU admission, 75 (27.4%) were MRSA and 83 (30.4%) were *A. baumannii*, which is the highest rate among Gram-positive and Gram-negative bacterium, respectively. When patients were discharged from the ICU, the rate of MRSA decreased from 27.4 to 20.4%; however, *A. baumannii* infection increased from 30.4 to 40.7%, whereas other bacterium did not change significantly (Table 4).

**Table 4:** Microorganisms isolation at the time of ICU admission and discharge

	ICU admission	ICU discharge
All microorganisms	1054 (100)	1040 (100)
Multidrug-resistant bacteria^a^	273 (25.9)	369 (35.5)
Methicillin-resistant *Staphylococcus aureus* ^b^	75 (27.4)	74 (20.1)
Vancomycin-resistant enterococci^b^	10 (3.6)	8 (2.2)
*Pseudomonas aeruginosa* ^b^	12 (4.4)	18 (4.9)
*Acinetobacter baumannii* ^b^	83 (30.4)	150 (40.7)
*Escherichia coli* ^b^	24 (8.8)	27 (7.3)
*Klebsiella oxytoca* ^b^	15 (5.5)	24 (6.5)
*Enterobacter cloacae* ^b^	5 (1.8)	3 (0.5)
*Proteus* species^b^	1 (0.4)	4 (1.1)
Other^b^	46 (16.8)	59 (16.0)
Non-multidrug-resistant bacteria^a^	781 (74.1)	671 (64.5)
*Staphylococcus epidermidis* ^c^	375 (45.7)	307 (45.8)
*Staphylococcus haemolyticus* ^c^	33 (4.2)	32 (4.8)
Methicillin-sensitive S*taphylococcus aureus* ^c^	49 (6.3)	51 (7.6)
*Enterococcus* species^c^	18 (2.6)	21 (3.1)
*Pseudomonas aeruginosa* ^c^	15 (1.9)	13 (1.9)
*Acinetobacter baumannii* ^c^	10 (1.3)	11 (1.6)
*Escherichia coli* ^c^	26 (3.3)	20 (3.0)
*Klebsiella oxytoca* ^c^	100 (12.8)	84 (12.5)
*Enterobacter cloacae* ^c^	13 (1.7)	12 (1.8)
*Proteus* species^c^	7 (0.9)	6 (0.9)
Others^c^	140 (17.9)	111 (16.5)

Data were presented by counts and percentage

^a^Percentage equals the counts of the item divided by all microorganisms

^b^Percentage equals the counts of the item divided by multidrug-resistant bacteria

^c^Percentage equals the counts of the item divided by non-multidrug-resistant bacteria

*ICU* intensive care unit

### Correlation between antimicrobial consumption and multidrug-resistant organisms

**Fig. 3:**
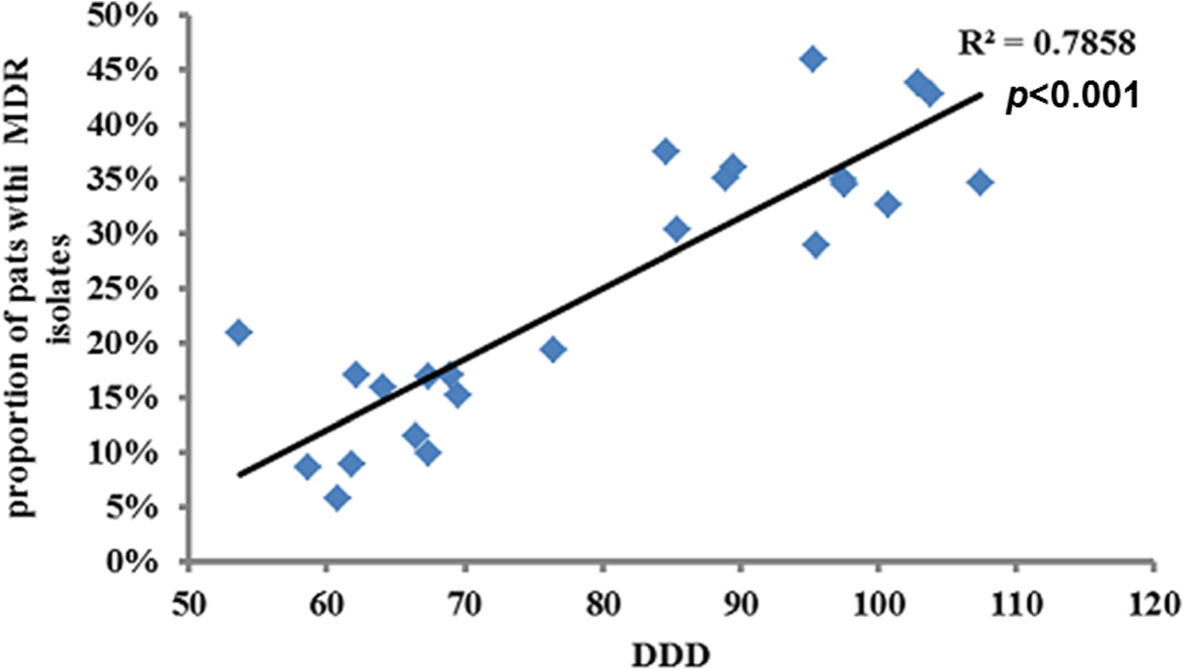
A significant positive relationship between the proportion of critically ill patient colonization or infection with MDRO at the time of ICU admission monthly and total antimicrobial consumption in our hospital monthly (R^2^ = 0.7858, *p* < 0.001)

As the antibiotic consumption decreased after antibacterial drug clinical application management, the proportion of colonization and infection with MDRO at the time of ICU admission correspondingly decreased. The total consumption of antibacterial agents each month during the study period had a significant positive relationship with colonization and infection with MDRO in critically ill patients at the time of ICU admission each month (R^2^ = 0.7858, *p* < 0.001) (Fig. 3).

**Fig. 4:**
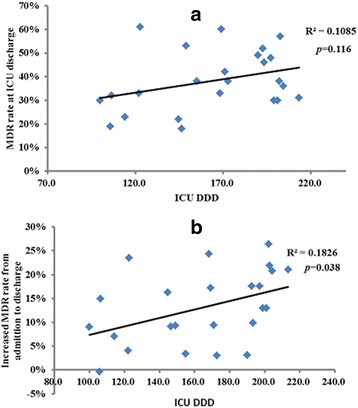
**a** There was no relationship between the DDD in the ICU and the MDRO rate at the time of ICU discharge (R^2^ = 0.1085, *p* = 0.116); **b** there was significant relationship between increased percentage of MDRO rate from the time of ICU admission to discharge from the ICU and DDD in the ICU (R^2^ = 0.2056, *p* = 0.038)

The DDD in the ICU may affect the MDRO proportion of critically ill patients at the time of ICU discharge. Therefore, we analyzed the relationship between these two factors. Although the MDRO rate decreased after the intervention, there was no significant relationship between the MDRO isolates and the DDD in the ICU (R^2^ = 0.1085, *p* = 0.116) (Fig. 4a). However, there was significant relationship between an increased MDRO rate from admission to discharge and the DDD in the ICU (R^2^ = 0.1826, *p* = 0.038) (Fig. 4b).

### Correlation between consumption of carbapenem and resistance of A. baumannii to carbapenem

*A. baumannii* was the most common MDRO in our study, and most *A. baumannii* are resistant to carbapenem. Therefore, we calculated the numbers of patients whose colonization or infection with *A. baumannii* were resistant to carbapenem at the time of ICU admission and discharge every month before and after the antimicrobial management. We also calculated the total carbapenem consumption in our hospital and in the ICU during the three-year study period. We analyzed the correlation between the consumption of carbapenem and the resistance of *A. baumannii* to carbapenem.

**Fig. 5:**
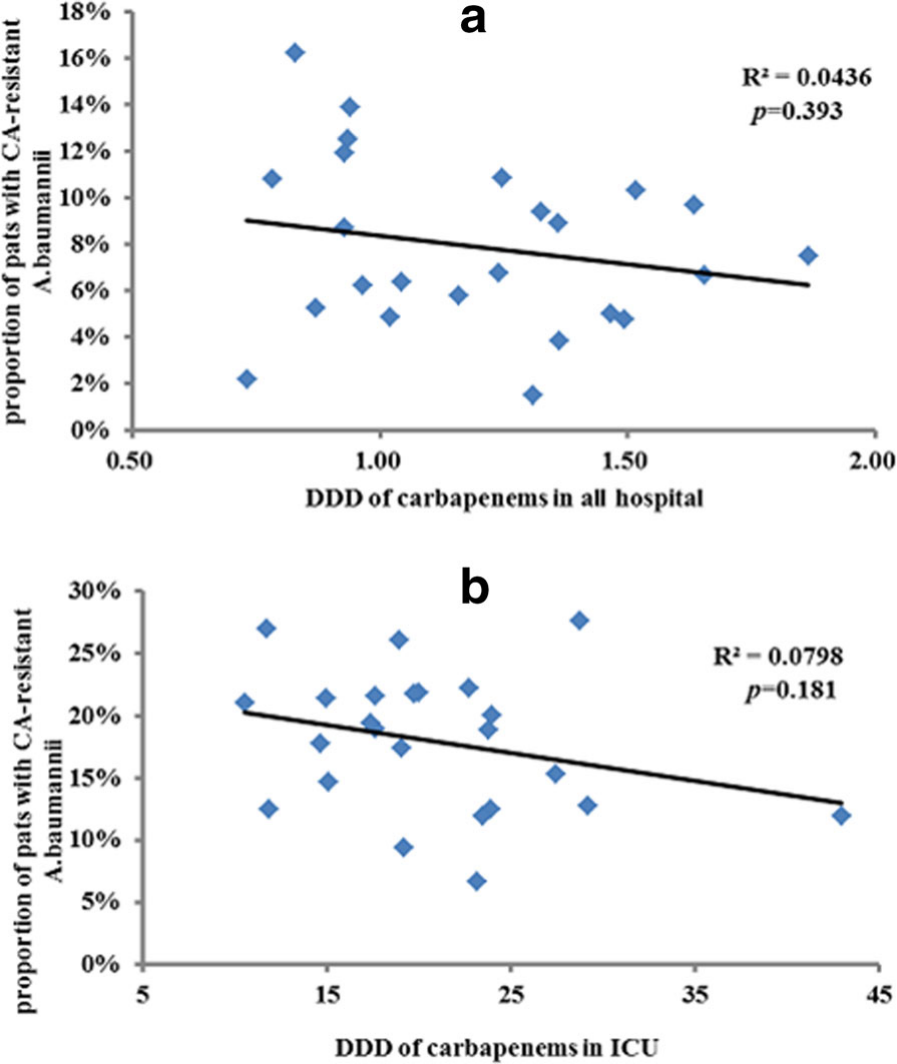
**a** There was no significant relationship between carbapenem consumption throughout the hospital and resistance of A. baumannii to carbapenem at the time of ICU admission (R^2^ = 0.0436, *p* = 0.393). **b** There was no significant relationship between carbapenem consumption in the ICU and resistance of A. baumannii to carbapenem at the time of ICU discharge (R^2^ = 0.0798, *p* = 0.181)

The proportion of patients colonized or infected with *A. baumannii* resistant to carbapenem at the time of ICU admission and discharge is presented in Fig. 5. The carbapenem consumption in our hospital and in the ICU during the three-year period decreased from 10.1 to 5.7% at the time of ICU admission and from 20.5 to 15.4% at the time of ICU discharge after the intervention. However, the DDD of carbapenem in the hospital and in the ICU did not significantly change after the intervention (1.33 ± 0.33 vs. 1.26 ± 0.28, *p* = 0.446 and 18.2 ± 5.4 vs. 23.2 ± 7.7, *p* = 0.573, respectively). There was no significant relationship between the consumption of carbapenem in the hospital and resistance of *A. baumannii* to carbapenem at the time of ICU admission (R^2^ = 0.0436, *p* = 0.393) (Fig. 5a). Similar results were found between the consumption of carbapenem in the ICU and the resistance of *A. baumannii* to carbapenem at the time of ICU discharge (R^2^ = 0.0798,* p* = 0.181) (Fig. 5b).

### The incidence of HCAIs before and after antimicrobial stewardship

During the intervention, we also monitored the incidence of ventilator associated pneumonia (VAP), catheter related blood stream infection (CRBSI) and catheter associated urinary tract infection (CAUTI), which were the most common of health-care-associated infections (HCAIs) in our ICU. We found that the incidence of VAP was significantly decreased after the intervention. However, the incidence of CRBSI and CAUTI did not change after the antimicrobial stewardship (Additional file 2: Table S2).

## Discussion

Infection with MDRO is frequently observed during clinical treatment [15–17], especially in critically ill patients [18]. Preventing infection with MDRO is very important to our clinic workers. To the best of our knowledge, this is the first study concerning the effect of the antimicrobial stewardship program of Chinese MOH on colonized or infected with MDRO in critically ill patients. In the present study, we found that DDD in the hospital and in most departments decreased significantly after the implementation of the antimicrobial stewardship.

Consequently, the MDRO isolation in critically ill patients correspondingly decreased when they admitted into ICU. However, we found a low relationship between DDD in the ICU and increased MDRO rate from ICU admission to discharge. Moreover, we did not find a positive relationship between DDD in the ICU and MRDO at ICU or between the DDD of carbapenem and carbapenem-resistant *A. baumannii*.

Antimicrobial stewardship can reduce the DDD in the entire hospital and in the majority of the departments. After the intervention, we found that the rational use of antibiotics had improved. In the preventative use of antibiotics in clean operation, for example, the percentage decreased from nearly 100 to 35%, which was in line with previous data [19]. Consequently, the DDD was decreased significantly from 96 to 65 in our hospital and was decreased in most departments (Table 2). Our results confirmed the previous results in China [20]. However, our DDD is still higher than the standard claimed by the policy. Therefore, we should conscientiously execute this policy to improve antibiotic use.

Antimicrobial stewardship can help to decrease the MDRO isolation in critically ill patients. Along with the decrease in antimicrobial consumption in our study, the proportion of colonization or infection with MDRO in critically ill patients decreased significantly to 13%. Furthermore, we found a good positive correlation between antibiotic consumption and MDRO in our hospital (Fig. 3). Previous studies demonstrated the association between antimicrobial use and MDRO detection [2–4, 21]. Bassetti et al. found a significant correlation between antibiotic consumption and increased resistance for *K. pneumonia* [21]. A recent study in China demonstrated that increased consumption of carbapenem may contribute to the development of resistance in *A. baumannii* to imipenem, meropenem, and other antimicrobials [4]. However, Most previous studies were demonstrated that with the increased consumption of antimicrobials, the MDRO increased correspondingly [2–4, 21–23]. There was only the some correlation between MDRO and antimicrobial consumption. Whether the decrease antimicrobial consumption could reduce the MDRO was not very clear. Interestingly, we found that the proportion of patients colonized or infected with MDRO decreased along with reduced consumption of antibiotics.

In some departments, such as the thoracic surgery department, the DDD did not decrease; however, the MDRO isolation rates in this department decreased significantly in critically ill patients. Several reasons may explain this decrease. First, DDD did not equal to rational application of antimicrobials. Moreover, the sample from such departments was small and could not achieve significance. Finally, MDRO are easily transmitted from patient to patient through hand contact with doctors, nurses and hospital workers [22, 23]. Additionally, critically ill patients were transferred several times to different departments before being initially admitted into the ICU. Therefore, the incidence of MDRO isolates in patients from different departments and throughout the hospital decreased, although DDD did not decrease.

Carbapenem-resistant *A. baumannii* is a serious problem in the clinic, especially in the ICU [24, 25]. Although the DDD of carbapenem did not decrease in the entire hospital and in the ICU, the carbapenem-resistant *A. baumannii* isolates decreased both at the time of ICU admission and at ICU discharge. Moreover, we did not find a good relationship between carbapenem consumption and carbapenem-resistant *A. baumannii*. Because previous studies have examined whether carbapenem restriction can reduce the rates of carbapenem-resistant *A. baumannii* and have had inconsistent findings [4, 26], further explorations are warranted to confirm these results.

Our study has several limitations. First, this study was performed in a single center. Because drug resistance rates vary among hospitals and units, the results may not be representative and reproducible in other institutions. However, in one hand, antibiotic overuse is common in China [10], and antimicrobial stewardship can improve the use of antibiotics. In another hand, the results of antimicrobial consumption in our study was in line with previous report [20]. Additionally, studies have demonstrated that the rational use of antibiotics could prevent antimicrobial resistance [27]. Therefore, we believe that our study can benefit other hospitals.

Second, we did not analyze the individual risk factors for colonization or infection with resistant microorganisms. Factors that would affect the MDRO isolation, such as hand hygiene, isolating the high-risk patients, and taking precautions, were not analyzed. However, during this pre-post study period in our hospital, we had taken executive infection control measures. We found that the compliance of hand hygiene did not significantly change in our department. Therefore, we believe that the decrease of DDD is an important factor for reducing the rate of MDRO. We also plan to investigate and analyze the risk factors concerning infection and MDRO.

Third, we did not screen the MDRO since rectal swabs, and the proportion of undetected MDRO colonizations could consequently be high. However, the policy of screen was the same during the two periods, therefore, it will not be influence our results hugely.

Finally, our study did not address the issue of antibiotic use and resistance in the community. The consumption of antimicrobials in agricultural industry may also promote antibiotic resistance [28, 29]. However, the MDRO isolation of patients who arrived from emergency department also decreased from 40 to 18% and may not affect our results.

## Conclusion

The present study has demonstrated that the antibacterial drug clinical application management established by China’s Ministry of Public Health can improve the rational use of antibiotics and reduce the antibiotic consumption. Consequently, with the decrease of DDD in the hospital, the proportion of colonization or infection with MDRO correspondingly decreased. There was a significant positive relationship between the proportion of all critically ill patients colonized or infected with MDRO at the time of ICU admission and the DDD of the all hospital. However, this relationship did not exist in the local department or DDD of the individual medication.

## Additional files

Additional file 1: Table S1. The category of antibiotics before and after antimicrobial stewardship. (DOCX 16 kb)
Additional file 2: Table S2. The incidence of HCAIs before and after antimicrobial stewardship. Incidence of VAP, CRBSI and CAUTI were defined as the number of VAP, CRBSI and CAUTI patients per 1000 ventilation days, per 1000 central venous catheter days and per 1000 urine-catheter days, respectively. (DOCX 15 kb)

## Abbreviations

CAUTI: Catheter associated urinary tract infection
CRBSI: Catheter related blood stream infection
DDD: Defined daily dose
ESBL: Extended-spectrum β-lactamase
HCAIs: Health-care-associated infections
ICU: Intensive care unit
MDRO: Multidrug-resistant organism
MIC: Minimal inhibitory concentration
MOH: Ministry of health
MRSA: Methicillin-resistant Staphylococcus aureus
VAP: Ventilator associated pneumonia
VRE: Vancomycin-resistant enterococci
WHO: World Health Organization
