# Supplementary material for: Antimicrobial stewardship of Chinese ministry of health reduces multidrug-resistant organism isolates in critically ill patients: a pre-post study from a single center
Source: BMC Infect Dis. 2016 Nov 25;16:704. doi: 10.1186/s12879-016-2051-8 (PMC5123232; doi:10.1186/s12879-016-2051-8)
Supplement: Additional file 1: Table S1. — The category of antibiotics before and after antimicrobial stewardship. (DOCX 16 kb) [file 12879_2016_2051_MOESM1_ESM.docx]

Table S1 The category of antibiotics before and after antimicrobial stewardship

| Category | Before management | After management | *p* value |
| --- | --- | --- | --- |
| All, n | 62 | 60 |  |
| Penicillins, n (%) | 7 (11.29) | 9 (15.00) | 0.544 |
| Cephalosporins, n (%) | 18 (29.03) | 18 (30.00) | 0.907 |
| Carbapenems, n (%) | 3 (4.84) | 3 (5.00) | 0.967 |
| Fluoroquinolones, n (%) | 8 (12.90) | 6 (10.00) | 0.615 |
| Aminoglycosides, n (%) | 4 (6.45) | 4 (6.67) | 0.962 |
| Macrolides, n (%) | 4 (6.45) | 4 (6.67) | 0.962 |
| Anti-fungi drugs, n (%) | 7 (11.29) | 7 (11.67) | 0.948 |
| Other, n (%) | 11 (17.74) | 9 (15.00) | 0.683 |
