# Supplementary material for: Antimicrobial stewardship of Chinese ministry of health reduces multidrug-resistant organism isolates in critically ill patients: a pre-post study from a single center
Source: BMC Infect Dis. 2016 Nov 25;16:704. doi: 10.1186/s12879-016-2051-8 (PMC5123232; doi:10.1186/s12879-016-2051-8)
Supplement: Additional file 2: Table S2. — The incidence of HCAIs before and after antimicrobial stewardship. Incidence of VAP, CRBSI and CAUTI were defined as the number of VAP, CRBSI and CAUTI patients per 1000 ventilation days, per 1000 central venous catheter days and per 1000 urine-catheter days, respectively. (DOCX 15 kb) [file 12879_2016_2051_MOESM2_ESM.docx]

Table S2 The incidence of HCAIs before and after antimicrobial stewardship

| HCAIs | Before management | After management | *p* value |
| --- | --- | --- | --- |
| VAP, n (‰) | 24 (12.38) | 19 (5.52) | 0.007 |
| CRBSI, n (‰) | 14 (4.02) | 19 (3.71) | 0.819 |
| CAUTI, n (‰) | 8 (2.04) | 11 (1.75) | 0.742 |

HCAIs: health-care-associated infections. CAUTI: catheter associated urinary tract infection. CRBSI: catheter related blood stream infection. VAP: ventilator associated pneumonia. Incidence of VAP, CRBSI and CAUTI were defined as the number of VAP, CRBSI and CAUTI patients per 1000 ventilation days, per 1000 central venous catheter days and per 1000 urine-catheter days, respectively.
